# Tobacco Poisoning and Its Effects upon the Eye-Sight

**Published:** 1879-09-20

**Authors:** A. W. Calhoun

**Affiliations:** Atlanta, Ga., Prof. of Eye, Ear, and Throat Diseases


### Southern Medical Record.

EDITORS:

T. S. POWELL, M.D. W. T. GOLDSMITH, M.D. R. C. WORD, M.D.

*R. C. WORD, M.D., Managing Editor.*

☞All Communications and Letters on Business connected with the RECORD must be addressed to the Managing Editor.

VOL. IX. ATLANTA, GA., SEPTEMBER 20, 1879. No. 9.

### ORIGINAL AND SELECTED ARTICLES.

### *TOBACCO POISONING AND ITS EFFECTS UPON THE EYE-SIGHT.*

BY A. W. CALHOUN, M. D., ATLANTA, GA., PROF. OF EYE, EAR, AND THROAT DISEASES.

[Read before the Medical Association of Georgia.]

That so palpable a fact as "tobacco blindness" should have so long remained unknown to the world, is indeed astonishing. Many physicians, and the people at large, are still skeptical, but among oculists, it has long been known that the excessive, and in many instances the very moderate, use of tobacco, will produce a train of symptoms, which, if let alone and the same cause be continued, will ultimately end in a partial or complete loss of sight, known as "tobacco amaurosis." That it does more or less damage in almost *every* instance, can be readily demonstrated by examining the throat of *any* smoker, in whom there will be found unmistakable evidences of slight or severe pharyngitis. It is not necessary to go far out of your way to find more than one person who justly attribute their unsteady gait, their tremulous hand and general "nervousness" *directly* to the habit of chewing or smoking, or both. I doubt not but that each one of you here can call to mind some friend or acquaintance, whose mental as well as physical strength has been seriously undermined, if not wrecked, by tobacco-poisoning. While damage may be expected from tobacco if used in any manner, it is mainly through smoking that the system is so filled with the poison that the optic nerve undergoes partial or complete atrophy, with corresponding blindness. There is, perhaps, nothing surprising in the fact that a large majority of one's acquaintances smoke, but there is something amazingly fearful in the quantity of tobacco used each day by an old smoker. The active principle, nicotine, is very abundant in tobacco, and is readily developed by burning. The smoker takes this poisonous principle into the mouth, the system becomes saturated, the delicate and sensitive nerves of vision become diseased, and their death, or atrophy, is the termination.

That men may, in many instances, harmlessly use tobacco for a long series of years, is no more an argument in opposition to its evil effects than that alcoholic stimulants can be taken, in exceptional cases, for a long life-time without serious detriment to the individual. That it injures the nervous system will scarcely be doubted by any one acquainted with its physiological effects, and that it takes the life of the nerve of sight, is now a fact beyond question.

The history of the following few cases, taken from the record of a large number in my possession, will give a fair insight into the nature of the disease under consideration.

*Case* 1.—Mr. B——, aged 50, is a large, vigorous man, in every way apparently in good general health, but for more than eighteen months has been gradually losing his sight. He is a farmer, and in the open air almost the entire day, and has been smoking for twenty years, but not till one and a half years ago did he notice any unpleasant effect from it. The quantity of tobacco daily consumed is difficult to estimate, but he is smoking his pipe from rising in the morning till he retires at night, with the exception of the time devoted to his meals. The quality is what he calls "first-class plug" tobacco, cut and rubbed into very small pieces, before being put into the pipe. The vision began failing, by his seeming to look through a thin cloud, which dimness has gradually increased, till finally, all objects appear covered by dense smoke. Accurately estimated, his vision fell to 10-50, and it was only with very strongly magnifying glasses that he could, even for a few moments, read very large newspaper type. There had never been the slightest pain, merely a gradual diminution of sight. At night he found the vision grew invariably more indistinct. The little floating bodies in the vitreous ("mouches volantes") were a great source of annoyance to him. With the aid of the ophthalmoscope, I found both optic discs decidedly anæmic, the right showing the white atrophic appearance much more than the left. The retina on each side was also more or less anæmic.

The treatment of this man was the immediate and complete cessation of the use of tobacco, and the administration of 1-20 gr. sulphate of strychnia three times daily.

It is now two years since the beginning of the treatment, and his vision has been raised from 10-50 to 10-12 (almost normal), and, with proper glasses, he reads ordinary print with ease and comfort.

*Case* 2.—Mr. W——, aged forty-two years, a banker, has smoked fifteen to twenty cigars a day for the last several years, but remains in perfect general health. For the past six months he has continually seen floating bodies (mouches volantes) before him, and sees everything through a mist. Towards night this mist increases in thickness, and very much obscures all objects. He has had no pain, but seeks advice because of increasing loss of sight. The ophthalmoscope shows an almost perfectly white papilla (atrophy of the optic nerve) in the left eye, and an anæmic condition of the nerve and retina in the right eye.

At once he stopped the use of tobacco, and took sulph. strychnia three times daily, and so regulated his business that he could remain a good portion of the day in the open air. The left eye remains about in the condition as in the beginning of treatment, but the right has been fully restored to its normal condition, and he is now constantly and actively engaged in his business.

*Case* 3.—Mr. D——, a machinist, aged about forty years, smokes his pipe ten or twelve times daily, and one cigar after each meal. When not smoking, he is chewing tobacco. He is a very early riser, and begins either smoking or chewing, sometimes both, before breakfast. Says he has noticed gnats or something like strings of beads (mouches volantes) floating before his eyes for one or two years, but only for the last ten or twelve months has everything appeared covered with a thin veil, which has by degrees grown thicker, till it now incapacitates him for work. No sort of glass has the slightest beneficial effect. He has had no pain, but, as in the majority of the other cases, says his vision is absolutely useless at night. The ophthalmoscope reveals a white atrophic nerve with anæmic retina in each eye. The vision is down to 10-40. The pupils contract and dilate slowly under the influence of light and shade.

He himself suspected the cause of his trouble, and, in part, proposed the treatment. He had in his pocket a few cigars, which he threw upon my table and has never touched tobacco in any shape or form from that day to this.

For a time I gave him hypodermically, 1-20 gr. strychnine once daily, gradually increasing the dose up to 1-6 gr. For some time past he has been taking it internally three times daily, in 1-30 gr. doses, and using electricity once daily or every other day. The vision has been restored to almost its normal condition, the increased blindness at night has been relieved, and the cloud before his eyes has disappeared.

*Case* 4.—A woman, Mrs. F——, who not only smokes her pipe freely, but is an immoderate "dipper" of snuff. This has been a habit with her for six or seven years. The cloudy vision, increasing towards night, the floating bodies, the white atrophic appearance of the optic disc, and anæmic retina are all present to a very marked degree, otherwise she is apparently very healthy.

She was, with difficulty, persuaded to quit the use of the pipe and *brush*, and put upon strychnia and electricity. The recovery in the course of a few months was perfect in one eye and partial in the other.

These suffice as a specimen of the most favorable class of cases of tobacco poisoning. Some had already complete atrophy of the optic nerve (amaurosis), and others, various other stages of the disease, in which treatment was of very little or no service. Perfect and incurable blindness from tobacco is of much more frequent occurrence than any one would dream of, except he be brought constantly in contact with eye diseases. Indeed, one writer says that not only does the optic nerve suffer such injury from smoking, but the brain itself has been affected by it to so great an extent in several instances that actual delirium tremens has resulted. Atrophy of the *auditory* nerve (nervous deafness) has also been traced to tobacco smoking.

From the cases here reported, the following will be noted as prominent and more or less invariable symptoms : Misty vision, the cloud gradually thickening, absence of pain, small bodies floating before the eyes, the vision becoming more blurred at night, the peripheral being much clearer than central vision. Some or all of these symptoms will be found in almost every well defined case. It has been said that the blindness begins in the right eye, but my experience does not bear me out in this observation.

Successful treatment depends upon total abstinence from tobacco. This is the first and most important step towards a cure. It is no easy matter to give up this habit, but I have been struck with the willingness with which patients abstain from its use, when once they are convinced that their vision is endangered. One or two cigars daily, or a few cigarrettes, suffice to keep up the poisoning, whereas, discarding it at once and entirely, will often, without further treatment, effect a perfect cure. Electricity aids materially in stimulating and strengthening the nerves, but sulphate of strychnia is the main reliance, in so far as medicines are concerned. I occasionally give it hypodermically in gradually increasing doses, but most frequently administer it in fluid or pill form, beginning with doses of 1-20 or 1-30 gr., slowly increasing to ¼ to 1-5 gr., given immediately before or after meals, that it may be digested with the food.

If by these notes of warning I may be the means of preventing in any one this seductive but dangerous indulgence, or of restoring sight to some dimmed eye, I shall feel fully repaid for my self-imposed task.

